# Supplementary figures and images for: Extracellular Vesicles—A Source of RNA Biomarkers for the Detection of Breast Cancer in Liquid Biopsies
Source: Cancers (Basel). 2023 Aug 30;15(17):4329. doi: 10.3390/cancers15174329 (PMC10487078; doi:10.3390/cancers15174329)

**The uncropped blots of Figure 1.**

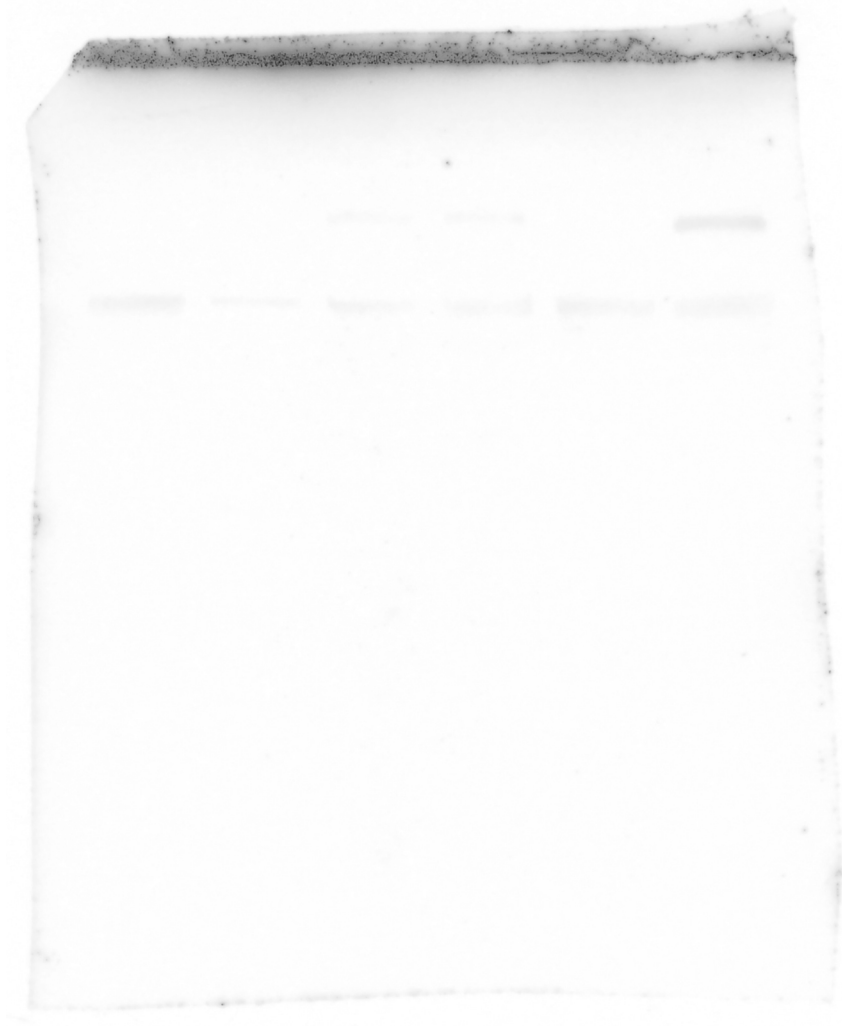

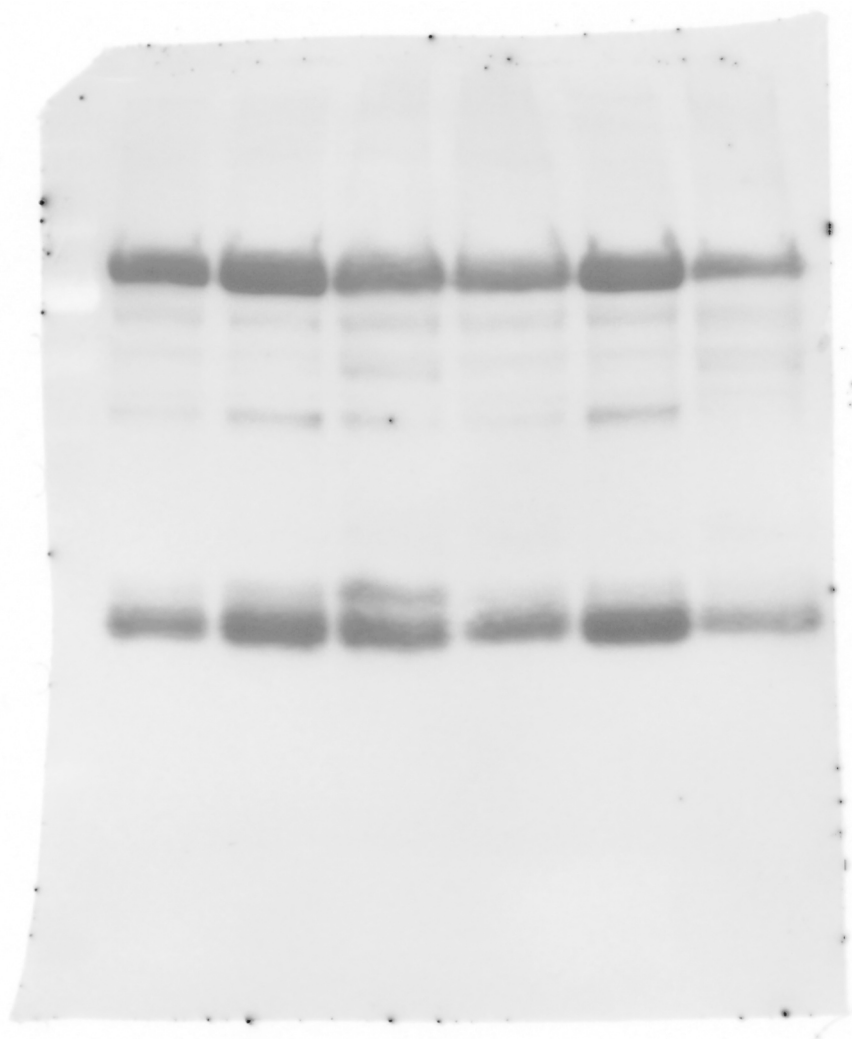

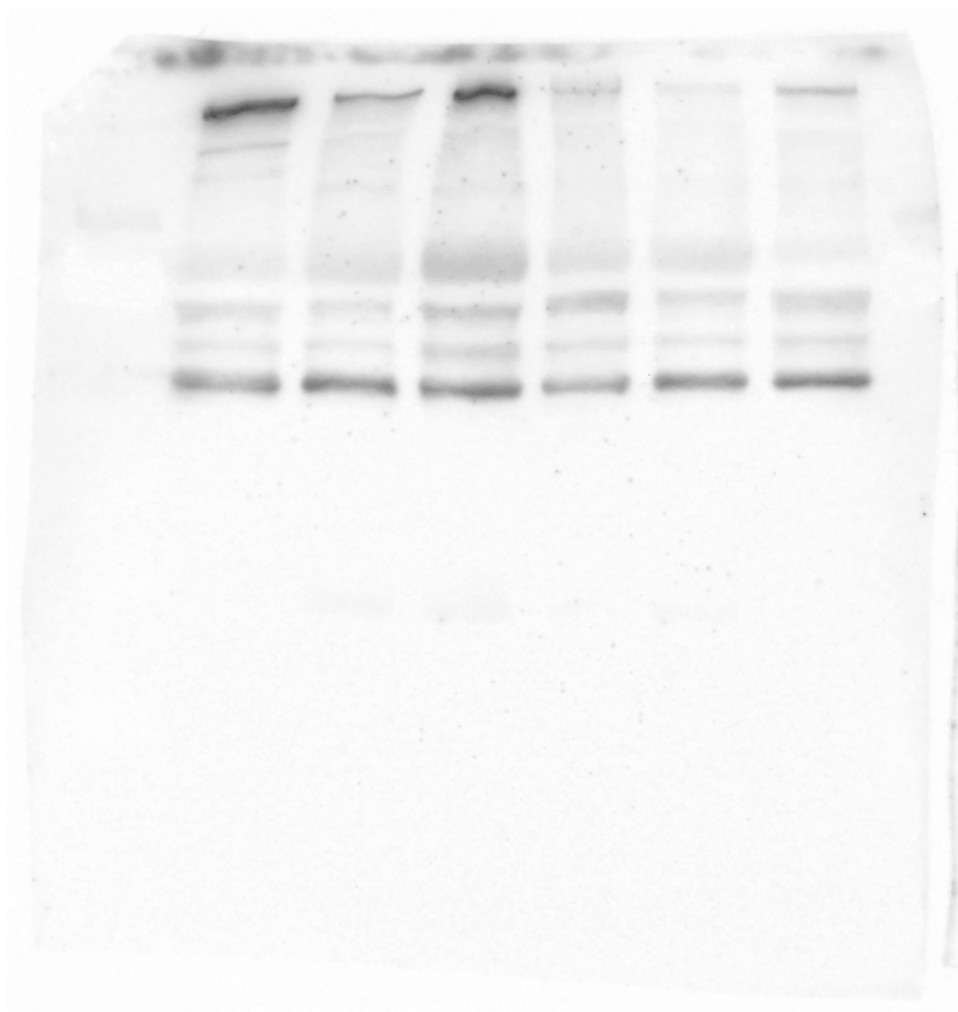

Supplement: Supplementary file 1 [file cancers-15-04329-s001.zip › File S1.pdf]
